# Computational Fluid Dynamics Modeling of Counter-Current Flow in Channels Separated by a Membrane

**DOI:** 10.3390/membranes16030109

**Published:** 2026-03-19

**Authors:** Akram Abdullah, Rathinam Panneer Selvam

**Affiliations:** Department of Civil Engineering, University of Arkansas, Fayetteville, AR 72701, USA; rps@uark.edu

**Keywords:** membrane, CFD, N-S equations, Darcy’s law, Poiseuille flow, counter-current flow

## Abstract

Several studies have investigated counterflow and concurrent flow in channels separated by a membrane to simulate mass transfer through membranes; however, few of them have used computational fluid dynamics (CFD). The current study aimed to numerically simulate and physically describe the distribution of pressure and velocity in counter-current flow by solving Navier-Stokes (N-S) equations in the channel and membrane pores (vertical channels). This is in contrast to most previous studies, in which the channel flow was simulated using N-S equations while ultra-filtration membrane flow was simulated using Darcy’s law. Consequently, the current study was executed using a CFD simulation to achieve several significant features: avoiding the execution of experimental tests, reducing the effort of model design and the expense and time consumption of fabrication, and facilitating the easy observation of variations in the pressure and the horizontal and vertical velocity for each point in the model. Two-dimensional CFD methods directly simulated the flow in channels and membrane pores to solve the N-S equations for each point in the whole domain, for which the velocity (horizontal and vertical) and pressure were calculated. In the current study, it was found that the pressure decreased from the inlet to the outlet of the channel, the horizontal velocity decreased from the inlet to the middle of the channel length and then increased to the outlet of the channel, and the vertical velocity decreased from the inlet to the middle of the channel length (L/2) with an upward direction (positive) and from L/2 to the outlet of the channel with a downward direction (negative). The analytical solution (1D model) was used to validate a numerical simulation (CFD) for the current study, but there were slight differences in the results between them. The results were perfectly explored and displayed the flow distribution patterns inside the channels and the membrane pores (vertical channels). The current study model represents the hemodialysis process.

## 1. Introduction

The characteristics of fluid flow in channels have been determined over several decades. Several studies have been performed using different assumptions and boundary conditions to study the flow in channels. The analytical solution for Poiseuille flows in solid channels has been reported by many researchers as follows [1]:

P = [12 × μ × u _mean_ × L]/(h^2^) (1)

where

P = Pressure at the inlet of the channel.
u _mean_ = Mean horizontal velocity at the inlet of the channel.
μ = Dynamic viscosity.
L = Length of the channel.
h = Height of the channel.

However, the flow between two porous parallel plates has been reported by few researchers. N-S equations have been solved to obtain a complete description of the fluid flow of two-dimensional incompressible steady-state laminar flow in a channel with a rectangular cross-section. One side of the cross-section, representing the distance between the two equally porous walls, is much smaller than the other. The solution leads to detailed expressions for the dependence of the velocity components and the pressure on the position coordinates, channel dimensions, and fluid properties [2]. A computer simulation was used to model the flow field of crossflow membrane filtration in a porous tube and shell system. Porous (permeable) wall flow is represented by Darcy’s law, which relates the pressure gradients within a flow stream to the flow rates through the permeable walls of the flow domain. The feed stream is modeled using N-S equations to represent viscous laminar Newtonian flow. The fluid dynamic model was developed for crossflow filtration; it is a robust, accurate, and cost-effective finite-element simulation scheme that is used to link N-S and Darcy equations in a solution [3]. The fluid dynamic model of a crossflow filtration tubular membrane was solved with numerical simulations using a finite difference scheme that was performed for laminar fluid flow in a porous tube with variable wall suction. The flow rates through the permeable wall were modeled using Darcy’s law, which relates the flow rate to the pressure gradient within a flow stream. The feed stream in the tube, which flows mainly tangentially to the porous wall, is modeled using N-S equations. The solution depends on both the Reynolds axial number and the filtration number [4]. The analytical solution of the Poiseuille flow between two porous parallel plates for two-dimensional incompressible fluid flow was reported. There was a crossflow (vertical velocity) along the y-direction due to the walls being porous. The analytical solution was found by applying the continuity equation and N-S equations with specific assumptions and boundary conditions. The solution depended on the cross-stream Reynolds number [1]. In recent decades, the flow in channels and membranes has been studied by several researchers; however, computational fluid dynamics (CFD) has only been used in a few studies to simulate fluid flow. The analytical solution was reported for the pressure drop in a rectangular channel with a constant wall velocity that is proportional to the trans-membrane pressure difference (constant wall permeability) and in a tube with porous (permeable) walls for constant wall permeability. A numerical CFD simulation for constant wall permeability was reported for comparison. The pressure drops in crossflow membrane modules are a function of the wall permeability, channel dimensions, axial position, and fluid properties [5]. The laminar fluid flow in a porous tube (crossflow filtration tubular membrane) was numerically simulated using CFD techniques. A two-dimensional numerical solution of the coupled N-S, Darcy’s law, and mass transfer equations was developed using the control-volume-based finite difference method. The researchers studied the effects of the geometrical dimensions, the required membrane surface area, the Reynolds number, and fouling on the performance of the membrane [6]. The crossflow membrane filtration was modeled through the relationship between hydrodynamics and the transfer of the flows across the membrane. From the model, some connecting variables were identified and established in this modeling work. By attaining these connections, membrane filtration can be optimized by adjusting the operating parameters. The FLUENT simulated model was in good agreement with the experimental results [7]. However, the actual micro-pore flow and the porous medium flow (Darcy flow) for various pressures and pore sizes (porosity and permeability) were simulated by using two-dimensional CFD models to solve N-S equations in all regions, including the pores, and to determine the pore flow average outlet velocity using the porous medium characteristics [8].

In membrane filtration processes where a combined free and porous flow occurs, N-S equations are used to model free flows, while Darcy’s law is used to model flows in porous media with a low porosity. A combination of free flow and flow through porous media occurs, and the continuity of the flow field regime across the interface between laminar flow and the porous region can be modeled by coupling Darcy’s law and the N-S equations.

Separation by membranes plays an important role in processing industries and in the medical sector, such as in hemodialysis. Many models have been developed to simulate mass transfer in hemodialysis. Counter-current flow was modeled using N-S equations, while membrane flow was simulated using Darcy’s law and the Kedem-Katchalsky (K-K) equation. Different computer models with finite elements were used for the simulation [3,9,10,11,12,13,14,15]. Stokes–Einstein’s equation was used for mass and momentum transfer [16]; however, Darcy’s law–Brinkman equations were used for flow across the membrane. Fiber in the dialyzer was modeled to calculate the mass transfer in the channels and membrane [17].

CFD has become an essential tool for analyzing complex transport phenomena in membrane-based systems, which are important for a wide range of applications from water purification to biomedical devices such as hemodialysis [18,19]. In processes such as hemodialysis, counter-current flow configuration—where two fluid streams move in opposite directions on either side of a semi-permeable membrane—is widely employed due to its potential for maximizing the concentration gradient and enhancing the mass transfer efficiency compared to those of concurrent flow [20,21].

The fundamental function of these systems relies on the transport of solutes (e.g., urea in blood) across the membrane, driven by diffusion (concentration gradient) and convection (pressure gradient). Accurately modeling this transport is complex, as it involves coupled flow phenomena in the open channels and through the membrane’s pores. A common approach in the literature has been to combine the N-S equations for channel flow with simplified models such as Darcy’s law or the K-K equations for flow through the membrane [3,9,16]. While this hybrid approach is practical, it depends on empirically determined membrane properties and may not fully resolve the detailed flow physics within the membrane’s pores themselves.

Recent studies have advanced the modeling of hollow fiber dialyzers, focusing on the module-scale performance and mass transfer [15,17,22]. However, a clear gap exists in the high-fidelity, pore-scale resolution of flow within the membrane. Many models treat the membrane as a continuum, neglecting the specific hydrodynamics inside the pores, which can critically influence phenomena such as back-filtration and the overall separation efficiency [23]. Directly solving the N-S equations throughout the entire domain, including for the membrane pores as in the current study, presents a computationally intensive but more physically comprehensive alternative. This approach eliminates the need for semi-empirical membrane models and can provide deeper insight into the local velocity and pressure fields that govern mass transfer.

Hollow fiber dialyzers are used for hemodialysis therapy. They have been examined by several authors. A hollow fiber dialyzer (membrane) is considered the main part of a hemodialysis device, and it has the following dimensions: 15–25 cm in length, 200 µm in inner diameter, and 8000–16,000 in number [12,13,15,17,21,23,24,25,26,27]. Membranes with an 8 nm pore size were used [16]. Membranes with a 40–80 nm pore size and a channel velocity of 1.67–2.78 mm/s were also studied. The results of these studies revealed an existing nanoscale reverse osmosis problem due to two opposite -running confined flows (countercurrent flow). To address this problem, the researchers recommended using two flows in the same direction (concurrent flow) instead of a counter-current flow [23]. However, counter-current flow comprising blood flow in the lower channel and fluid flow (dialysate) in the upper channel was studied, and the researchers found that counter-current flow resulted in a 20% increase in removal compared to concurrent flow [20]. The transmembrane pressure and the total filtration rate were compared between counter-current and concurrent flow in channels separated by a membrane [21]. The membrane height (thickness: 30 µm) and channel flow (300–500 mL/min) were used to examine the flow in dialyzers [15,28]. Reverse osmosis happens when the hydraulic transmembrane pressure cannot overcome the flow, resulting in back-filtration, especially in channels with a 10 nm height. By decreasing the diameter of the hollow fibers, the number of fibers will increase, and consequently, the contact filtration area will increase. This will improve the filtration efficiency. Predicting and optimizing the flow through these microscopic channels is key to making dialysis faster and is critical for designing more efficient hemodialysis equipment [23].

The current study examined the flow distribution patterns inside channels and membrane pores to explore the process of hemodialysis. It used membranes with pores of a pore size (vertical channels) of 4 µm and 15 µm in height. In hemodialysis, the membrane height is 30 µm and depends on concentration differences (diffusion), not pressure differences (convection) [15]. Blood and dialysis solutes in hemodialysis are present on both sides of the membrane in the absence of a pressure difference. The hemodialysis separation process works with a concentration difference (difference in diffusion rate and molecular size) for a porous membrane thickness of 10–100 µm [29].

In the current study, counter-current flow was modeled in channels separated by a membrane in order to solve the continuity and the N-S partial differential equations. Finite difference methods in CFD were used to find the velocity and pressure at any point in the entire flow domain, including the channels and membrane pores (vertical channels).

Therefore, the current study presents a detailed 2D CFD model of counter-current flow in channels separated by a membrane, with a specific focus on resolving the flow within the membrane pores. Unlike previous works that relied on Darcy’s law, the current model solves the full N-S and continuity equations simultaneously across the entire flow domain—the channels and the membrane pores. This methodology leverages modern computational capabilities to provide a direct and detailed visualization of the pressure and velocity distribution. The primary objectives are to physically describe the distribution of pressure and velocity that develops under counter-current conditions and to demonstrate the advantages of a full-domain CFD solution for the design and optimization of membrane contactors, ultimately saving the time and cost associated with extensive experimental prototyping.

The main objective of the current study was to numerically simulate and physically describe the counter-current flow in channels separated by a membrane by solving N-S equations in the channel and membrane pores (vertical channels) instead of solving Darcy’s law for membrane pores, which requires the execution of a physical experiment. Consequently, this approach will save time and cost. The current study used CFD to solve the N-S equations and to find the flow parameters for the whole domain (channel and vertical channels in the membrane). The use of CFD is characterized by the following advantages:

1. CFD can precisely model and visualize the flow conditions with a higher accuracy compared to measuring with physical equipment, and it eliminates the need for the execution of experimental tests, which are challenging to perform in tiny channels and membranes.
2. CFD reduces the expense and time consumption associated with physical modeling and experimental tests, and it identifies potential problems, such as backflow, before the manufacture of costly physical modules.
3. CFD helps to easily observe variations in the pressure and the horizontal and vertical velocity through computer modeling; these features are very difficult to examine using real experiments.

## 2. Materials and Methods

### 2.1. Model Concept

The membranes were fabricated using direct 3D printing with dimensions of 75 µm × 75 µm × 15 µm (height) and pores of approximately 3.73 µm in diameter and very high porosities that reached up to 60%, to be used for specific applications in microfluidics [30].

**Figure 1:**
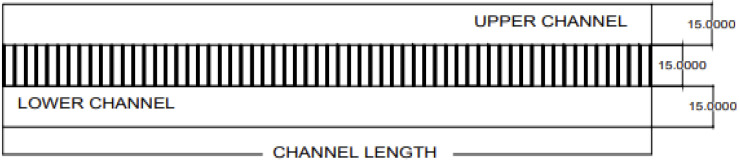
Model of counter-current flow in channels separated by a membrane (dimensions in µm).

To examine the membrane efficiency and performance, the membrane was modeled for counter-current flow in channels. The model was proposed with the dimensions and properties shown in Figure 1.

The current study examined the model shown in Figure 1 by producing transmembrane pressure differences to produce flow through the membrane pores (vertical channels), with the following properties:

1. The upper channel is 15 µm in height (the flow is from right to left) (backward).
2. The membrane (vertical channel) height is 15 µm with a membrane pore size of 4 µm and a wall thickness of 1 µm.
3. The lower channel is 15 µm in height (the flow is from left to right) (forward).
4. The channels and membrane are 6–301 µm in length.
5. Two opposite running flows are used in the model, with an axial mean velocity of 1 m/s, a viscosity of 3 × 10^−4^ kg/(m s), and a Re of 1/300, with a channel and membrane length of 6–301 µm.
6. Counter-current fluid was used with the same viscosities.

We studied the two-dimensional steady-state incompressible laminar flow of a fluid in counter-current channels with a rectangular cross-section separated by a membrane, using the solution of the continuity and N-S equations.

Why did the current study use 2D flow and not 3D flow? Our model used a height (h) = 15 µm, length (L) = variable, and width (w) >>> height (h); this agrees with the justification reported in the literature [1,2]. The current study considered 2D flow to simulate the case with CFD by decreasing the number of grid points and the time required to run the program.

The general continuity equation for 2D incompressible fluid flow has been previously reported [31].

∂u/∂x + ∂v/∂y = 0 (2)

The general N-S equations for 2D incompressible fluid flow have also been reported [31].

ρ (u ∂u/∂x + v ∂u/∂y) = −∂p/∂x + μ (∂^2^u/∂x^2^ + ∂^2^u/∂y^2^) + ρg_x_ (3)

ρ (u ∂v/∂x + v ∂v/∂y) = − ∂p/∂y + μ (∂^2^v/∂x^2^ + ∂^2^v/∂y^2^) + ρg_y_ (4)

The physical properties and a description of the current study are provided in Table 1 and Figure 1.

#### 2.1.1. Governing Equations

In two-dimensional flow, the governing equations are as follows:

(I) The continuity equation for 2D flow (x, y) is

∂u/∂x + ∂v/∂y = 0(5)

(II) The N-S equations for 2D flow (x, y) are

1/ρ (∂p/∂x) = ν (∂^2^u/∂x^2^ + ∂^2^u/∂y^2^) (6)

1/ρ (∂p/∂y) = ν (∂^2^v/∂x^2^ + ∂^2^v/∂y^2^) (7)

#### 2.1.2. Boundary Conditions

**Figure 2:**
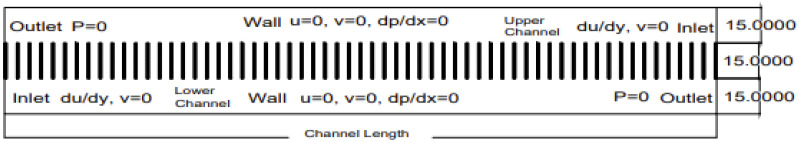
Counter-current flow in channels separated by a membrane—boundary conditions.

Boundary conditions were adopted as mentioned in the following computational procedure and are shown in Figure 2.

1. At the inlet of the channels, a fully developed laminar profile was considered, i.e., Poiseuille flow. At x = 0: u = u(y) = 1.0 m/s (assumed), v = 0, and p was calculated from u.
2. At the outlet of the channels, a fully developed profile was assumed. At x = L, P = 0.
3. At the outlet of the channels, all derivatives in the flow direction were set to zero; du/dx = 0.
4. At the walls of the channels, there were no momentum fluxes crossing the boundary. At y = h: ∂u/∂y = 0 or u = 0, ∂ v/∂y = 0 or v = 0.

The solutions to the governing equations were modeled by using CFD. CFD simulations helped to predict how changing the geometry of these microscopic channels—such as the size, shape, and distribution of membrane pores—affects these transport processes. CFD is a useful tool in the modeling of the flow in channels and membranes. A CFD simulation was used to study the flow and mass transfer for hollow fiber membrane modules utilized in devices. The simulation modeled the N-S equations for the flow in channels and Darcy’s law for the membrane as a porous medium [21,22,26,27,32]. Linking the channel flow (upper and lower channels) and membrane flow (ultra-filtration) was difficult because the boundary conditions for the membrane surface could not be assumed [23]. CFD models have been presented in which micro-pores are modeled using N-S equations to solve the flow in all regions, including the pores [8]. The analytical solutions for two fluids flowing in parallel or counterflow through passages separated by a permeable wall as a 1D model have also been derived [28].

However, the model in the current study examined a 2D steady incompressible laminar Newtonian fluid to solve continuity and N-S equations, using FDM in CFD to determine and calculate the pressure and horizontal and vertical velocity at any point in the whole model, including the channels and membrane pores (vertical channels). The physical description of the channel flow and the membrane (ultrafiltration) flow is governed by the N–S equations. The velocity components and the pressure on positional coordinates, channel dimensions, and fluid properties were obtained. The CFD approach was adopted here as a useful tool for flow visualization, as it provides a cost-effective and accurate means to find flow characteristics within a whole-domain model.

The current study used in-house CFD software, which was programmed by Prof. Selvam, R.P., to solve N-S equations, find the pressure and velocity for the whole domain (channel and membrane), and visualize the results with the help of Tecplot. The in-house CFD software was designed to achieve residual thresholds < 10^−5^; horizontal velocity, pressure, and vertical velocity value convergence within a tolerance < 1%; a relaxation factor (RF) = 1; and 300–9000 iterations depending on the channel length. Most previous studies developed models to find the solutions to N-S equations, Darcy’s law, and K-K equations. The velocity and pressure in the channel flow are described by N-S equations, while the filtration flow is described by (K-K) equations or Darcy’s law to predict the flow through a membrane. The actual micro-pores can be described by using two-dimensional CFD models to solve the N-S equations in all regions, including the pores [8]. However, to the best of the authors’ knowledge, the current study is the first study that has used CFD to model N-S equations of 2D counter-current flow in channels separated by a membrane to find the velocity and pressure for the whole domain. The Poiseuille flow Equation (1) was used to calculate the pressure along the channel without a membrane to explain the difference in the results for channels with a membrane, obtained using CFD. The 2D domain was gridded with a uniform mesh (square grid spacing of 0.25 µm × 0.25 µm) of 181 × 1205 in the y and x directions and ~218,100 nodes for simulations (for 45 µm (2 channels and a membrane) × 60 pores (301 µm)). The CFD program was run until the steady-state solution was obtained after a number of iteration cycles. This approach was used because it is difficult to experimentally measure the pressure and velocity distribution at different locations.

### 2.2. Computational Procedure

The steady-state N-S equations were approximated using the finite difference procedure. Since the Reynolds number was less than 1, the convection term was not considered in the current solver. The momentum and pressure equations were solved using the successive over-relaxation (SOR) procedure on a non-staggered grid. Since an equal grid spacing was used all around the domain, the diagonal term for U and V (Ap term) was the same everywhere, and hence, it was used to form the pressure Poisson equation as follows:

∇. U′ − ∆P/Ap = 0(8)

Here, U′ is the velocity vector obtained by solving the momentum equations. The above equation, with P = 0 at the outlet and dP/dx = 0 on the wall, was used to solve for P. Once P was solved to the required convergence, the velocities were updated as follows:

U = U′ − (dP/dx)/Ap(9)

V = V′ − (dP/dy)/Ap(10)

The current study examined the flow in the channel as Poiseuille flow to find the pressure and horizontal velocity variation along the channel length, which remained constant for the horizontal velocity and decreased at a constant rate for the pressure. However, the case was different for counter-current flow in channels separated by a membrane. To understand this case, the current study used the CFD software program and Tec-plot visualization to calculate the pressure and the horizontal and vertical velocity; then, Excel and Tecplot were used to visualize the results.

## 3. Results

**Figure 3:**
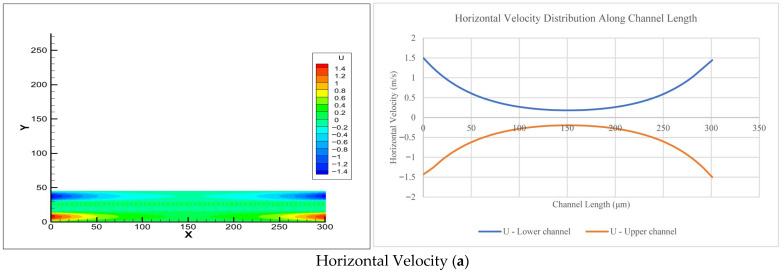

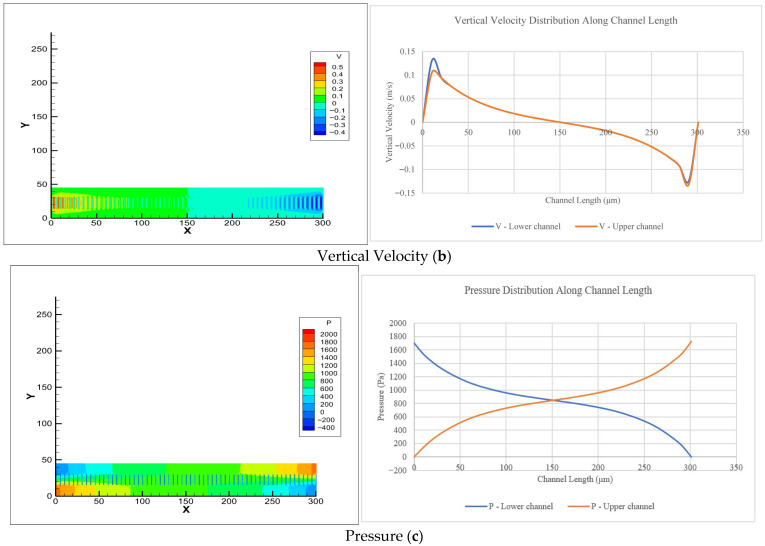
Horizontal velocity, vertical velocity, and pressure for a channel length of 301 µm. Where: X = Length of channel and membrane in μm. Y = Height of channel and membrane in μm. U = Horizontal velocity in m/s. V = Vertical velocity in m/s. P = Pressure in Pa.

The current study gathered the CFD results for different channel lengths (6 µm to 301 µm). Figure 3 shows the horizontal velocity, vertical velocity, and pressure variation at the h/2 of the upper and lower channels for a counter-current in a channel length of 301 µm, with visualization in Excel and Tec-plot as the output of the CFD software.

Figure 3 shows that the horizontal velocity decreased from the inlet velocity (1.5 m/s) to 0.194 m/s (representing 13% of the maximum velocity (1.5 m/s)) at L/2 of the channel length, and it then increased to the outlet velocity (1.5 m/s). The vertical velocity decreased from the inlet to L/2 of the channel length (0.148 m/s to 0) with an upward direction from the inlet velocity, and from L/2 of the channel length to the outlet (0 to 0.148 m/s) with a downward direction. The pressure decreased from the inlet pressure (1725 Pa, representing 36% of the maximum pressure (4816 Pa = Poiseuille pressure)) to the outlet pressure (0), and not at a linear rate. The CFD results do not follow the Poiseuille flow pattern.

For the channels (6–301 μm in length), the CFD results show that the horizontal velocity decreased from the inlet to some intermediate distance (channel middle = L/2) and then increased back to the outlet of the channel. The decrease in horizontal velocity increased as the channel length increased. The vertical velocity decreased as the channel length increased but did not do so at a constant rate. The direction of the vertical velocity was upward from the inlet to the middle of the channel length (L/2) and changed direction downward from the middle (L/2) to the outlet of the channel length. The flow in the membrane pores was due to the pressure difference, which produced a vertical velocity that created the flow through the membrane pores. The pressure increased as the channel length increased but did not do so at a constant rate. This increase was required to create flow through the channels and membrane pores.

**Figure 4:**
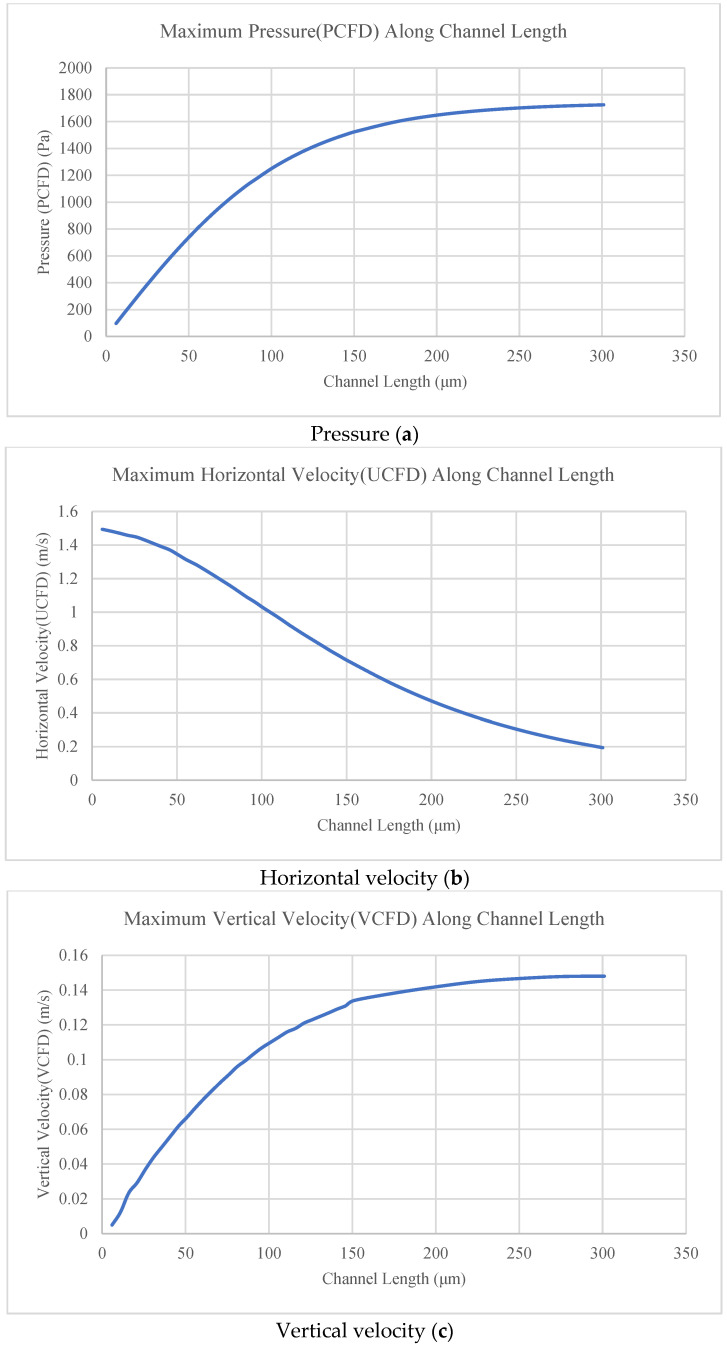
Pressure, horizontal velocity, and vertical velocity along channel length. Where: U = maximum horizontal velocity at L/2 of the channel length and h/2 of the channel height. P = maximum pressure at the inlet and h/2 of the channel height. V = maximum vertical velocity at the inlet and h/2 of the channel height.

The pressure (P_CFD_) and the velocity (U_CFD_, V_CFD_) were obtained from the computer model for the channel length (6–301 μm), as shown in Figure 4.

Figure 4a shows that the pressure increased as the length of the channels increased. Figure 4b shows that the horizontal velocity at L/2 decreased as the length of the channels increased. Figure 4c shows that the vertical velocity increased as the length of the channels increased, but it did not do so at a constant rate, eventually plateauing. From Figure 4, the horizontal velocity (U), vertical velocity (V), and pressure (P) can be calculated for any channel length (6–301 μm) at the middle of the channel (L/2) for U and at the inlet of the channel for V and P.

In the current study, the counter-current flow did not follow the Poiseuille flow equation because of the existence of the pores in the membranes, which took part of the horizontal velocity and the pressure in the form of vertical velocity to generate flow through the pores of the membrane. The transmembrane pressure from the CFD results ranged from 97 Pa (0.14 psi) to 1725 Pa (2.5 psi) for channel lengths of 6–301 µm. This is classified as a microfiltration process [29].

### 3.1. Non-Dimensional Pressure and Velocity Variation

**Figure 5:**
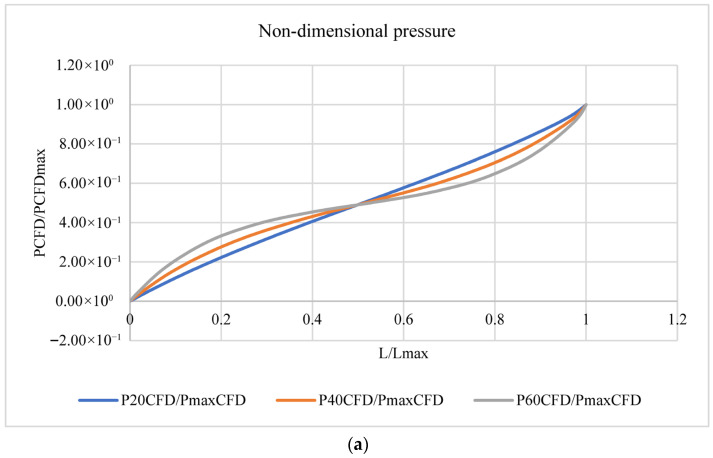

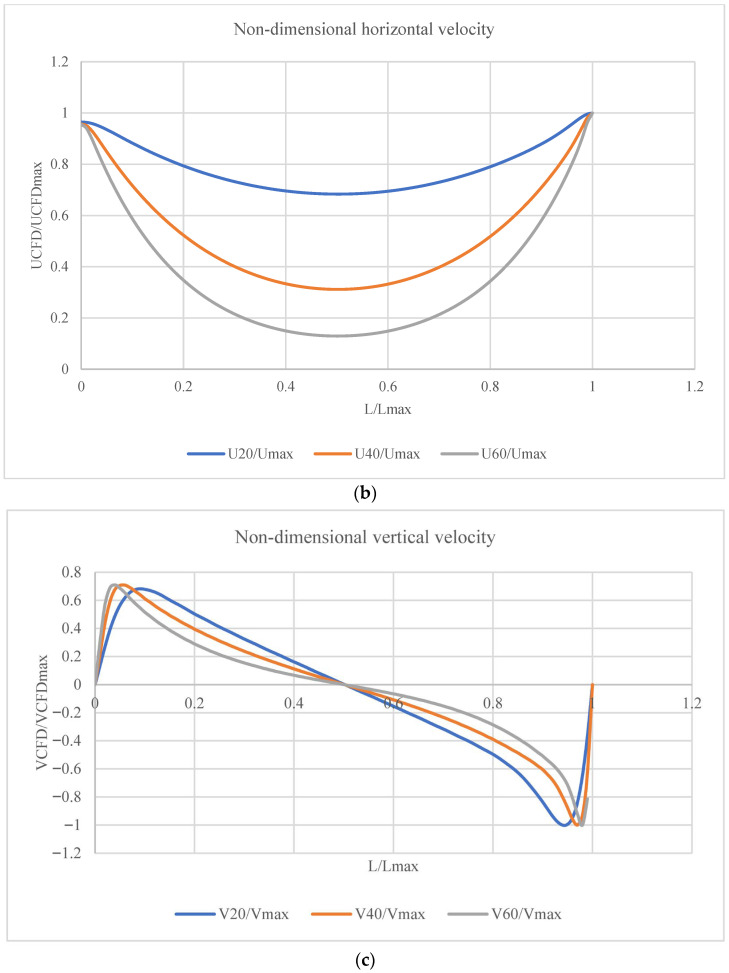
Non-dimensional pressure (**a**), horizontal velocity (**b**), and vertical velocity (**c**) with non-dimensional length of the channel.

The pressure, horizontal velocity, and vertical velocity for the counter-current flow at the inlet of channels with 20 (101 µm), 40 (201 µm), or 60 (301 µm) pores are presented and plotted in a non-dimensional form to show the effect of the existence of a membrane. These results are shown in Table 2 and Figure 5.

Table 2 shows that the horizontal velocity decreased while the pressure and vertical velocity increased as the length of the channels increased. For example, the horizontal velocity (UCFD) was only 68% of the inlet U max and the pressure (PCFD) was only 78% of the analytical P for the channel with 20 pores (101 µm), while these values were 13% and 36% for the channel with 60 pores (301 µm), as previously mentioned.

P_Analytical_ can be calculated using Equation (1). Figure 5 shows the non-dimensional pressure, horizontal velocity, and vertical velocity, where

P = pressure variation at the inlet of the channel.
U = horizontal velocity variation at L/2 of the channel length.
V = vertical velocity variation along the channel.

Figure 5a shows that the non-dimensional pressure increased as the non-dimensional channel length increased. Figure 5b shows that the non-dimensional horizontal velocity decreased as the non-dimensional channel length increased, along with the channel length (L). Figure 5c shows that the non-dimensional vertical velocity changed from the inlet to the outlet and that this change was not linear. It decreased until reaching the middle of the channel length (L/2) (positive/upward direction) and then changed its direction (negative/downward direction) to the outlet of the channel.

### 3.2. Pressure and Velocity Variation Along the Membrane Pores (Vertical Channels)

**Figure 6:**
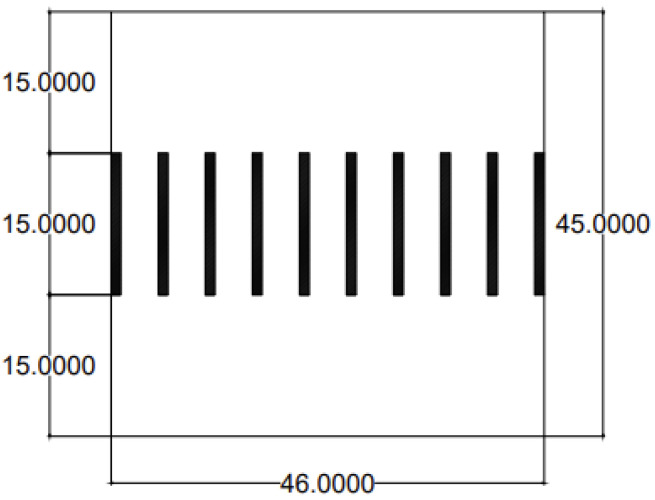
Counter-current flow in channel (9 pores = 46 µm) separated by membrane (dimensions in µm).

**Figure 7:**
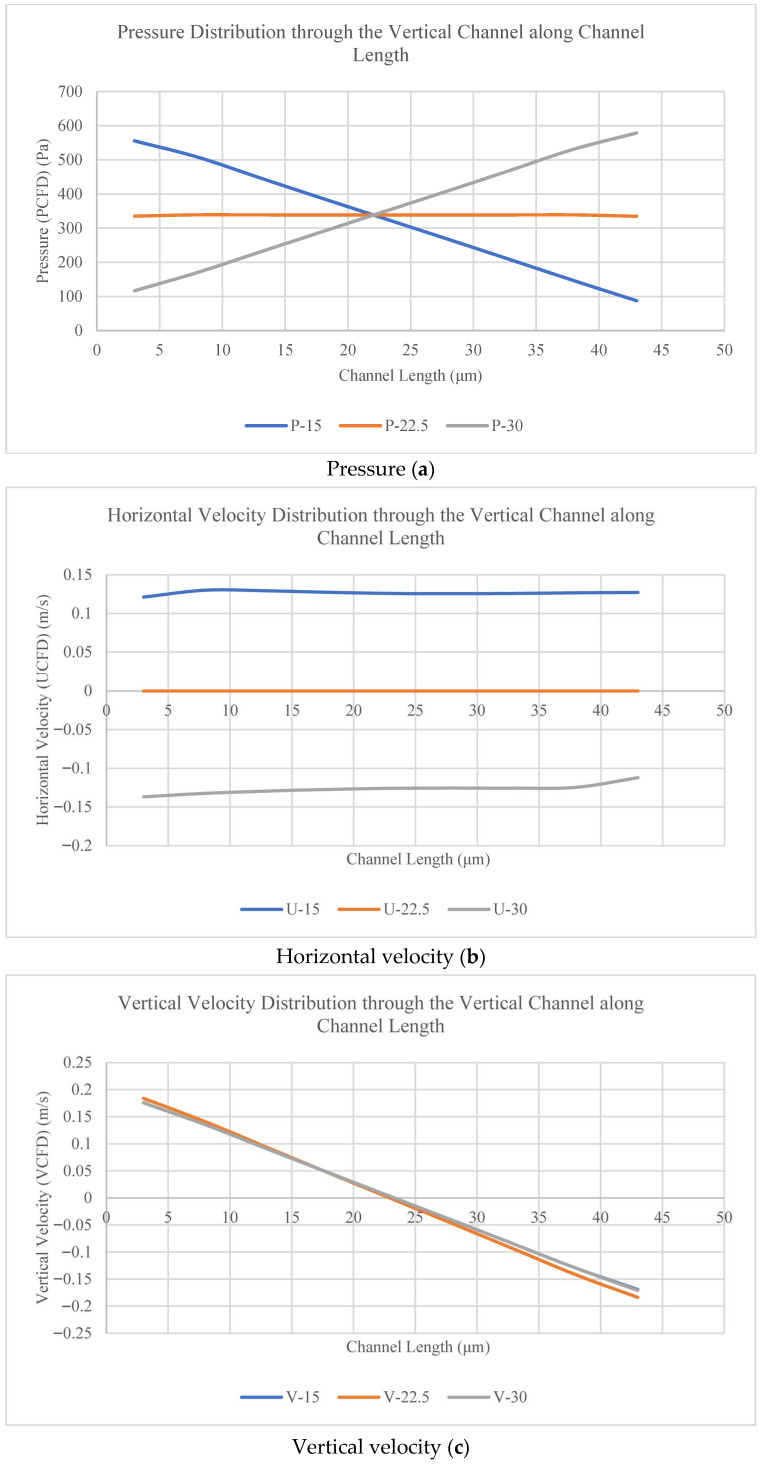
Pressure (**a**), horizontal velocity (**b**), and vertical velocity (**c**) in the membrane pores (vertical channels) along the channel length.

At the inlet (y = 15 μm), middle (y = 22.5 μm), and outlet (30 μm) of the membrane pores (vertical channels) (for x = 3, 8, 13, 18, 23, 28, 33, 38, 43 µm), the pressure, horizontal velocity, and vertical velocity for the channel with nine (46 μm) pores are plotted to show the variation inside the membrane pores (vertical channels), as shown in Figure 6 and Figure 7.

Figure 7a shows that the pressure in the membrane pores (vertical channels) at the inlet and outlet decreased along the length of the channel, while it remained constant at the middle height of the vertical channels. Figure 7b shows that the horizontal velocity in the membrane pores (vertical channels) at the inlet and outlet remained constant along the length of the channel, while it was almost zero at the middle height of the vertical channels. Figure 7c shows that the vertical velocity in the membrane pores (vertical channels) followed the same pattern as in the horizontal channels (upper and lower channels). It decreased until the middle of the channel length (L/2) (positive/upward direction) and then changed its direction (negative/downward direction) to the outlet of the channel.

## 4. Validation of CFD Numerical Solution with 1D Analytical Solution

There are many methods for validating simulation model results. One of the methods is an analytical solution, while another is experimental procedures. Most studies prefer an analytical solution over experimental procedures because of the cost and time associated with the latter. The current study used a 1D analytical solution to validate the numerical CFD results [28]. The current study modeled the counter-current flow as shown in Table 1 and Figure 1. The permeability (k), porosity, and hydraulic permeability (Lp) parameters used in the 1D analytical solution were calculated as shown in Table 3 [28].

**Figure 8:**
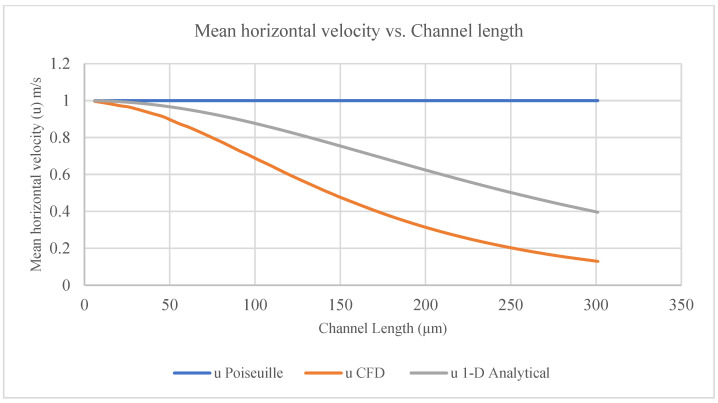
Comparison of the mean horizontal velocity calculated for the channel length between the models.

**Figure 9:**
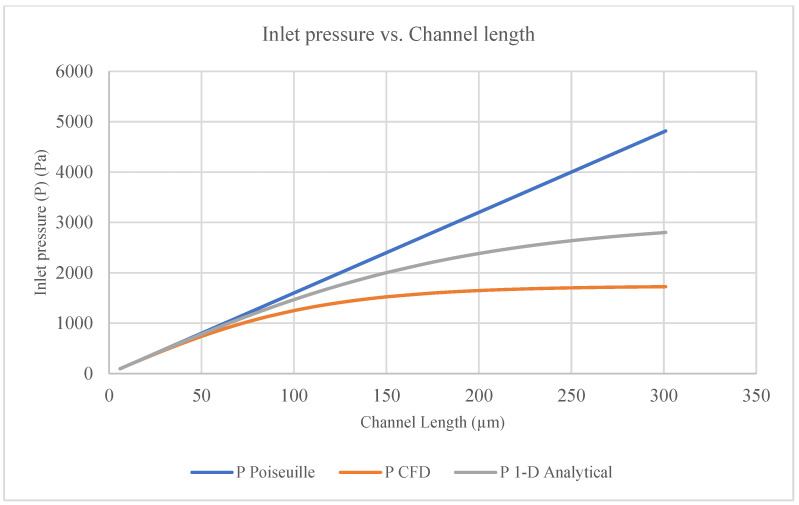
Comparison of the inlet pressure calculated for the channel length between the models.

For the current study, the CFD model’s numerical results were compared with the 1D analytical model results and the Poiseuille flow between two solid parallel plates [28]. Figure 8 shows a comparison of the mean horizontal velocity calculated for the channel length (6–301 µm) between the models. Figure 9 shows a comparison of the pressure calculated for the channel length (6–301 µm) between the models.

There were differences between the results of the 1D analytical model and the CFD model used in the current study [28]. These differences were due to the boundary conditions and assumptions and due to differences in the equations that were used to solve the flow in the channel. The CFD model used continuity and N-S equations for the flow in the channel and membrane pores (vertical channels), while the 1D analytical model used a continuity equation for the flow in the channel and Darcy’s law for the flow through the membrane [28]. The 1D analytical model computed a single average velocity per cross-section, while the 2D CFD model calculated lateral velocity variations at a grid-cell level. The 1D analytical model results for a permeable wall were close to the CFD numerical results for a membrane, which provides substantial confidence in the current study results [28].

## 5. Analysis and Discussion

Most previous studies used N-S equations to model channel flow and Darcy’s law to model transmembrane ultrafiltration flow (membrane flow). Such studies found that the drops in channel pressure, the transmembrane pressure, the channel velocity, and the ultrafiltration velocity were nearly linear. However, the current study used N-S equations to model both channel flow and membrane pore flow (vertical channels). The differences in the pressure between the lower and upper channels decreased until L/2 (which produced the minimum horizontal velocity and zero vertical velocity) and increased to the maximum until L. This difference in pressure produced horizontal velocity in the channel (horizontal channel), but the differences did not demonstrate a constant rate. The horizontal velocity decreased from the maximum at the inlet (0) to the minimum at L/2 and then increased to the maximum at the outlet (L). Mass transfer through the membrane pores occurs due to pressure differences between the upper and lower channels. The pressure is at its maximum at the inlet of the channels. This means that the maximum pressure occurs between the inlet of the upper channel and the outlet of the lower channel, and vice versa between the outlet of the upper channel and the inlet of the lower channel. The differences in pressure produce vertical velocity in the membrane pores (vertical channels) from the lower channel to the upper channel, and this velocity decreases from the inlet to L/2 of the channel length (upward direction—positive) as the differences in pressure decrease. The differences in the pressure will produce vertical velocity in the membrane pores (vertical channels) from the upper channel to the lower channel, and this velocity decreases from the inlet to L/2 of the channel length (downward direction—negative) as the differences in pressure decrease. This maximum pressure difference results in high mass transfer flux through the membrane pores (vertical channels). This provides an explanation for why the current study used counter-current flow rather than concurrent flow. According to the results of the current study, the existence of a membrane changes the properties of the flow from Poiseuille flow between two rigid plates to flow in channels separated by a membrane.

Counter-current flow could improve the uniformity of the mass flux distribution in mass transfer, as the flow rate decreases from the inlet to some intermediate distance and then increases back to the outlet [17,28,32,33]. The mass transfer was determined for both concurrent and counter-current flow, and the counter-current flow was found to be more efficient and associated with a 20% increase in removal compared to concurrent flow [20,21,34,35]. In contrast, concurrent flow is recommended instead of counter-current flow [23].

In the current study, as shown in Table 4, the membrane pore size was 4 µm (vertical channels), and the height (thickness) was 15 µm. The pores in the membrane were 3.75 µm; according to the IUPAC (International Union of Pure and Applied Chemistry), such pores are classified as macropores (>50 nm (0.05 µm)), and a process is considered an ultrafiltration process when the membrane pores are 2–100 µm (porous membrane) for ∆P = 1 to 5 bar (10,000 Pa–50,000 Pa). The transmembrane pressure in this study, according to the CFD results, ranged from 97 Pa (0.14 psi) to 1725 Pa (2.5 psi) for channel lengths of 6–301 µm. Thus, this is classified as a microfiltration process [29]. In hemodialysis, a membrane height of 30 µm was reported, and this height depends on the concentration differences, not on the pressure differences [15,28]. Blood and dialysis solutes in hemodialysis are present on both sides of the membrane with the absence of a pressure difference, and it works with a concentration difference (difference in diffusion rate and molecular size) for porous membrane thicknesses of 10–100 µm [29].

There is vertical velocity in the channel as a result of a membrane’s existence; this velocity is responsible for the flow through the membrane pores. An increase in the transmembrane pressure (=upper/lower channel inlet pressure − lower/upper channel outlet pressure) causes an increase in the amount of ultrafiltration. At the interface between the channel and membrane surface, the current study found that the direction of the vertical velocity was upward to L/2 of the channel length, and then the direction changed to downward from L/2 to L of the channel length. This means that the flow in the membrane pores is due to the vertical velocity produced from the transmembrane pressure differences. The pressure and the horizontal and vertical velocity variations in the channel are not linear, but the pressure inside the membrane pores (vertical channels) is linear because these channels have a small length (15 µm) and they follow Poiseuille flow. The horizontal velocity decreased from the channel inlet to L/2 of the channel and increased to the outlet. The horizontal velocity decreased as the channel length increased, up to the length that resulted in a horizontal velocity of almost zero. This phenomenon causes issues in microfluidic devices due to backflow and stagnation.

## 6. Conclusions

In the current study, counter-current flow did not follow the Poiseuille flow equation because of an increase in the number of pores in the membrane with channel length. The pores in the membrane take part of the pressure and horizontal velocity in the form of vertical velocity to create flow in these pores. The flow pattern in the current study (2D model) can be summarized as follows, and as shown in Figure 3 and Figure 4:

1. The horizontal velocity decreases from the inlet to the middle length of the channel (L/2) and increases to the outlet. It decreases in the middle of the channel (L/2) as the length of the channel increases.
2. The pressure decreases from the inlet to the outlet of the channel. It increases as the length of the channel increases.
3. The vertical velocity decreases until the middle of the channel length (L/2) (positive/upward direction) and changes its direction (negative/downward direction) to the outlet of the channel. It increases as the length of the channel increases.

The results of the current study can be used to improve the efficiency of devices and decrease the time required to treat a patient’s blood, especially when using hemodialysis, if counter-current flow is utilized in a channel separated by a membrane. For counter-current dialysate flow, the authors of [10,15,20] suggest that using different channel heights (one channel for blood and the other channel for dialysate) is more efficient. If the best combination of channel heights (lower and upper) is utilized, it would increase the contact time between blood and dialysate (solute) through the membrane by increasing the vertical velocity through the membrane pores (the vertical channel); this would in turn decrease the time that the patient needs to remain under hemodialysis, thus decreasing the level of suffering and pain. The CFD model results slightly matched the 1D analytical model results, which provides some confidence in the current study’s results and opens the door to more research on the use of CFD to simulate N-S equations for 3D flow [28].

## 7. Current Study Contributions

Most previous studies used CFD to model channel flow by solving N-S equations and transmembrane ultra-filtration flow (membrane flow) by solving Darcy’s law or K-K; however, this method requires the execution of physical experiments to determine certain parameters, such as the permeability. These studies found that, as the channel pressure drops, the transmembrane pressure, channel velocity, and ultra-filtration velocity are nearly linear. To the best of the authors’ knowledge, the current study is the first study to use CFD to model two-dimensional counter-current flow in channels separated by a membrane. This was achieved by solving N-S equations in the channels and membrane pores (vertical channels) to find the velocity and pressure at any point in the whole domain, as these values are difficult to experimentally measure. Consequently, this approach represents a reliable tool that can save cost and time. Instead of conducting expensive and time-consuming physical experiments in the manufacture of microfluidic devices, the presented method can be used to test and optimize hundreds of different device designs.

## Figures and Tables

**Table 1:** The current study’s input parameters.

Parameter	Unit	Values	Notes
Channel (lower and upper) height (h_c_)	µm	15	h_c_ = 1.50 × 10^−5^ m
Membrane height (h_m_)	µm	15	h_m_ = 1.50 × 10^−5^ m
Length of the channel (lower and upper) and membrane (L)	µm	6–301	L = 6.0 × 10^−6^ m − 3.01 × 10^−4^ m
Density (ρ)	Kg/m^3^	1000	Water at 4 °C
Dynamic viscosity (μ)	[(Pa·s) =(N·s/m^2^) = (kg/m.s)]	3.00 × 10^−4^	
Kinematic viscosity (ν)	(m^2^/s)	3.00 × 10^−7^	ν = μ/ρ
Reynold’s number (Re)	-	3.33 × 10^−3^ = 1/300	Re = ρ u L/μ = u L/ν
Maximum horizontal velocity (u_max_)	m/s	1.5	Assumed at inlet
Average horizontal velocity (u_mean_)	m/s	1.0	u_mean_ = 2/3 u_max_

**Table 2:** Non-dimensional values for horizontal velocity, vertical velocity, and pressure.

Channel Length (No. of Pores)	V_inlet_-_CFD_	U_middle-CFD_	U_middle-CFD_/U_max inlet_	P_CFD_	P_ANALY_	P_CFD_/P_ANALY_
101 µm (20)	0.110	1.0252	0.683	1258	1616	0.778
201 µm (40)	0.142	0.467	0.311	1650	3216	0.513
301 µm (60)	0.148	0.194	0.129	1725	4816	0.358

Where U_max inlet_ (m/s) (1.5 m/s) = maximum horizontal velocity at the inlet of the channel without a membrane (Poiseuille flow). U_CFD_ (m/s) = maximum horizontal velocity at L/2 of the channel length with a membrane. V_CFD_ (m/s) = maximum vertical velocity at the inlet of the channel with a membrane. P_CFD_ (Pa) = maximum pressure at the inlet of the channel with a membrane. P_Analytical_ (Pa) = analytical pressure at the inlet of the channel without a membrane (Poiseuille flow).

**Table 3:** 2D Permeability (k), porosity (ϕ), and hydraulic permeability (Lp) for 2D channels.

No.	References	Equation	Description
1	[31]	K = h^2^/12	Permeability/2D channel = 1.88 × 10^−11^ m^2^
2	[30]	Porosity (ϕ) = volume of voids/total volume	Porosity (ϕ)/2D channel = 0.64
3	[28]	Lp = ∆Q/L × w × p	Hydraulic permeability/2D channels = 5.16 × 10^−5^ m/Pa.s

**Table 4:** Comparison between the current study and recent related studies.

	Current Study	References [15,28]
Channel height (µm)	15	45 (upper channel), 75 (lower channel)
Membrane thickness (height) (µm)	15	30 µm
Channel length (µm)	6–301	15,000–25,000, used (24,400)
Pore size (R) (µm)	4.0	-
Fiber inner diameter (D) (µm)	-	200 No 8000–16,000
Q blood	-	300 mL/min
Q dialysis	-	500 mL/min
Forward velocity (inlet)	1 m/s	-
Backward velocity (inlet)	1 m/s	-
Forward pressure (inlet)	variable	-
Backward pressure (inlet)	variable	-
Transmembrane pressure	97 Pa (0.14 psi)–1725 Pa (2.5 psi)	concentration difference
Filtration process	microfiltration	ultrafiltration

## Data Availability

The data presented in this study are available from the corresponding author upon request.

## Nomenclature

CFD	computational fluid dynamics
N-S	Navier-Stokes
K-K	Kedem-Katchalsky
P	pressure (N/m^2^ = kg/m.s ^2^ = Pascal)
U, u	horizontal velocity (m/s)
V, v	vertical velocity (m/s)
L	channel length (m)
h	channel height (m)
D	dimension (m)
Re	Reynolds number
Q	flow rate (m^3^/s)
u, v, w	velocity components in the x, y, and z directions (m/s)
g _x, y, z_	gravity components in the x, y, and z directions (m/s^2^)
**Greek letters**	
µm	micrometer
ρ	density (Kg/m^3^)
μ	dynamic viscosity (kg/m.s)
ν = μ/ρ	kinematic viscosity (m^2^/s)
